# Excessive Daytime Sleepiness is associated with Incident Dementia in Community‐Dwelling Older Adults: A 10‐year Longitudinal Study

**DOI:** 10.1002/alz70861_108772

**Published:** 2025-12-23

**Authors:** Xiaowen Zhou, Guangshi Zhang, Qianhua Zhao, Ding Ding

**Affiliations:** ^1^ Institute of Neurology, Huashan Hospital, Fudan University, Shanghai China; ^2^ National Center for Neurological Disorders, Huashan Hospital, Fudan University, Shanghai China; ^3^ National Clinical Research Center for Aging and Medicine, Huashan Hospital, Fudan University, Shanghai China

## Abstract

**Background:**

In this 10‐year longitudinal study, we verified the hypothesis that the excessive daytime sleepiness (EDS) was associated with incident dementia in community‐dwelling older adults, and examined whether this association is influenced by the amyloid pathology.

**Method:**

We included 618 dementia‐free participants with baseline and follow‐up interviews (2010‐2024) from Shanghai Aging Study. At baseline, EDS was measured by the Epworth Sleepiness Scale (ESS), and plasma phosphorylated tau‐217 (*p* ‐tau217) was measured using a single‐molecule immune‐array assay. Cognitive function was evaluated using a battery of neuropsychological tests including Mini‐Mental State Examination (MMSE) at baseline and follow‐up. A consensus clinical diagnosis of incident dementia was ascertained based onmedical, neurological, and neuropsychological examinations.

**Result:**

At baseline, 39 of 618 participants (6.3%) reported EDS (ESS≥10). Participants with EDS demonstrated significantly lower MMSE scores compared to those without EDS (27.67 vs. 28.35, *p* < 0.05). Higher ESS scores were significantly associated with reduced baseline MMSE scores (β = ‐0.050, 95% CI: ‐0.099 to ‐0.002; *p* = 0.041), and this relationship was moderated by baseline plasma *p* ‐tau217 levels. Over a median follow‐up of 10 years, 56 participants developed dementia. Baseline EDS was associated with an elevated risk of incident dementia (hazard ratio [HR] = 2.02, 95% CI: 1.06‐3.83; *p* = 0.032), adjusted by age, sex and education years. Significant association between EDS and incident dementia was found in individuals with low baseline *p* ‐tau217 level (< 0.295 pg/ml) (HR = 3.74, 95% CI: 1.40‐9.99; *p* = 0.008), but not in those with high *p* ‐tau217 levels (HR = 1.45, 95% CI: 0.65‐3.24; *p* > 0.05).

**Conclusion:**

EDS may serve as an early behavioral marker of incident dementia in community‐dwelling older adults, particularly among those without significant amyloid pathology. Early recognition of EDS may thus improve risk stratification and support timely interventions to reduce future dementia risk.

